# Magnetoencephalographic alpha band connectivity reveals differential default mode network interactions during focused attention and open monitoring meditation

**DOI:** 10.3389/fnhum.2014.00832

**Published:** 2014-10-15

**Authors:** Laura Marzetti, Claudia Di Lanzo, Filippo Zappasodi, Federico Chella, Antonino Raffone, Vittorio Pizzella

**Affiliations:** ^1^Department of Neuroscience, Imaging and Clinical Sciences, “G. d’Annunzio” UniversityChieti, Italy; ^2^Institute for Advanced Biomedical Technologies, “G. d’Annunzio” UniversityChieti, Italy; ^3^Department of Psychology, Sapienza UniversityRome, Italy

**Keywords:** meditation, mindfulness, magnetoencephalography, default mode network, resting state networks, brain rhythms

## Abstract

According to several conceptualizations of meditation, the interplay between brain systems associated to self-related processing, attention and executive control is crucial for meditative states and related traits. We used magnetoencephalography (MEG) to investigate such interplay in a highly selected group of “virtuoso” meditators (Theravada Buddhist monks), with long-term training in the two main meditation styles: *focused attention* (FA) and *open monitoring* (OM) meditation. Specifically, we investigated the differences between FA meditation, OM meditation and resting state in the coupling between the posterior cingulate cortex, core node of the Default Mode Network (DMN) implicated in mind wandering and self-related processing, and the whole brain, with a recently developed phase coherence approach. Our findings showed a state dependent coupling of posterior cingulate cortex (PCC) to nodes of the DMN and of the executive control brain network in the alpha frequency band (8–12 Hz), related to different attentional and cognitive control processes in FA and OM meditation, consistently with the putative role of alpha band synchronization in the functional mechanisms for attention and consciousness. The coupling of PCC with left medial prefrontal cortex (lmPFC) and superior frontal gyrus characterized the contrast between the two meditation styles in a way that correlated with meditation expertise. These correlations may be related to a higher mindful observing ability and a reduced identification with ongoing mental activity in more expert meditators. Notably, different styles of meditation and different meditation expertise appeared to modulate the dynamic balance between fronto-parietal (FP) and DMN networks. Our results support the idea that the interplay between the DMN and the FP network in the alpha band is crucial for the transition from resting state to different meditative states.

## Introduction

Recently, the neural correlates of meditation states and traits have been increasingly studied in cognitive and affective neuroscience (Cahn and Polich, 2006; Lutz et al., 2008; Raffone and Srinivasan, 2010). Such growth of interest has been supported by several findings about the salutary effects of meditation on physical and mental health, as related in particular to mindfulness based programs (e.g., Chiesa and Serretti, 2010; Keng et al., 2011). Moreover, neuroimaging findings have clarified the role of brain structures and processes involved in meditation and mindfulness based training (e.g., Cahn and Polich, 2006; Chiesa and Serretti, 2010).

Several conceptualizations of meditation practice have underpinned a central role for attention and cognitive control skills (Lutz et al., 2008; Malinowski, 2013a,b). These skills are crucial for the development and maintenance of mindfulness, the intentional and non-judgmental awareness of the fields of experience in the present moment, such as about perceptual, thought and feeling contents, that, in turn, leads to therapeutic outcomes and wellbeing effects (Kabat-Zinn, 1994; Wallace and Shapiro, 2006; Malinowski, 2013a). Meditation practices can be usefully classified into two main styles—*focused attention* (FA) and *open monitoring* (OM)—depending on how the attentional processes are directed (Cahn and Polich, 2006; Lutz et al., 2008). In the FA (“concentrative”) style, attention is focused on a given object in a sustained manner, and thus emphasizes sustained attention and attention regulatory skills. The second style, OM meditation, involves the monitoring of any content of ongoing experience, and thus emphasizes mindfulness rather than FA and attentional control (Sumedho, 1994; Goldstein and Kornfield, 2001).

In this framework, the interplay between brain networks related to attention and control processes, and self-related processing appears fundamental for the understanding of meditation and mindfulness skills (Malinowski, 2013b). Indeed, mind-wandering and self-related processes occupy a large part of mental activity of human beings (Killingsworth and Gilbert, 2010): imagining future events, thinking about something different from what is currently being done, are mental states that frequently occur in everyday life (Killingsworth and Gilbert, 2010). It is not surprising, then, that mind-wandering parallels the brain’s mode of operation that is associated with the recruitment of the so called Default Mode Network (DMN; Raichle et al., 2001). The DMN is one of the most robust among the Resting State Networks (RSNs), and entails the posterior cingulate cortex, the medial prefrontal cortex, the posterior lateral parietal/temporal cortices, and the parahippocampal gyrus (Raichle et al., 2001; Buckner et al., 2008; Watanabe et al., 2013). These areas have shown to be co-activated during passive mental states (e.g., task-unrelated cognition).

Another network that appears relevant for meditation and mindfulness skills is the Fronto-Parietal control network (FP). The FP network includes many regions identified as supporting cognitive control and decision-making, such as lateral prefrontal cortex, middle frontal gyrus, anterior insula/frontal operculum, anterior cingulate cortex, and anterior inferior parietal lobule (Vincent et al., 2008). The FP network also supports internally vs. externally focused goal-directed cognition by coupling with either the default or dorsal attention network (Christoff et al., 2009; Spreng et al., 2010; Spreng and Schacter, 2012). This functional interplay between the DMN and FP represents a model for goal-directed cognition that might significantly contribute to the understanding of the functional mechanisms underlying meditation.

Functional MRI (fMRI) studies have so far shown an enhancement of BOLD functional connectivity between the nodes of the DMN and executive control brain areas at rest and during meditation practice in selected meditator populations (Brewer et al., 2011). The recruitment of the DMN during meditation has been hypothesized to signal the involuntarily drift of attention away from the focus of meditation towards mind wandering (Hasenkamp et al., 2012; Tang et al., 2012). Nevertheless, more recent work has shown that activation of the DMN might serve for adaptive functions beyond rumination and mind wandering (Ottaviani et al., 2013). Moreover, evidence exists for a differential coupling of the DMN with other brain regions in different meditation styles (Xu et al., 2014), thus indicating a possibly more complex involvement of the DMN in meditation.

A long tradition of studies has investigated the functional role of brain rhythms by electroencephalography (EEG) or magnetoencephalography (MEG), and, in particular, the functional correlate of the alpha rhythm has been long debated. Oscillations in the alpha band (8–12 Hz) have been classically interpreted as the functional correlate of drowsiness, being of larger amplitude during e.g., eye closed (Berger, 1929), supporting the idea of alpha power as related to an “idling” state. Later evidence pointed out that an increase of alpha power is associated to deactivation of task-irrelevant brain areas, whereas a power decrease in alpha is associated to their activation (Pfurtscheller, 2003). This idea was advanced into an alpha-inhibition hypothesis, which suggests that alpha synchronization may reflect top-down control processes (Klimesch, 1996, 1999; Klimesch et al., 2007). Opposite evidence exists for task-related increase in alpha power for high level cognitive processes such as those elicited by mental calculation, mental imagery, or internally driven attention (e.g., Hari et al., 1997; Cooper et al., 2003; Kounios and Beeman, 2009). Along the same line, recent evidence from alpha phase-synchronization/phase-coherence, which is hypothesized to represent a mechanism for short and long range communication in the brain (Lachaux et al., 1999; Varela et al., 2001; Fries, 2005; Palva et al., 2005; Engel et al., 2013), suggests a direct role for alpha band synchronization in the functional mechanisms of attention and consciousness (Palva and Palva, 2007; Knyazev, 2013). In this framework, it is not surprising that several studies have shown a link between DMN structures and power and phase synchronization in the alpha frequency range with concurrent EEG-fMRI (Laufs et al., 2003a,b; Mantini et al., 2007; Jann et al., 2009; Michels et al., 2010; Sadaghiani et al., 2010, 2012; Knyazev et al., 2011) and MEG (de Pasquale and Marzetti, 2014).

Brain rhythms, as measured by EEG and MEG, have also been studied to the specific aim of disclosing the impact of meditation practices. These studies, revealed a high heterogeneity of the frequency specific signatures of brain changes induced by meditation both in the same or in different traditions (Cahn and Polich, 2006; Ivanovski and Malhi, 2007; Nolfe, 2011). In this framework, evidence exists for the Individual Alpha Frequency (IAF; Klimesch, 1999) to be lowered as a consequence of intensive meditation training in Saggar et al. (2012). Due to IAF lowering, in those subjects, part of the standard alpha band was indeed pertaining to an individualized beta band, in which the authors found training induced power modulations. This finding might implicitly suggest that the heterogeneity reported in the literature might also be a consequence of the same nomenclature used for frequency bands that might potentially not fully overlap across different studies.

Furthermore, it should be noted that the great majority of EEG/MEG studies have investigated frequency specific connectivity changes at the level of electrodes/sensors and their results cannot be directly related to specific brain areas or brain networks due to volume conduction confounds (Schoffelen and Gross, 2009).

The recent development of methods to study ongoing functional connectivity at brain level with MEG, opens the way for reconciling the view on intrinsic activity as expressed by specific spatial brain modes, i.e., RSNs, and alpha band role in attention and consciousness (Engel et al., 2013), offering a direct window into the high complexity of brain information processing (de Pasquale et al., 2012; Larson-Prior et al., 2013). On the basis of such development, here we studied MEG functional connectivity at the level of brain sources to investigate DMN interactions in meditation. Specifically, we used a phase-coherence based approach that estimates the degree of linear coupling/synchronization between oscillatory signals of distant neuronal ensembles. We hypothesize that DMN internal coupling as well as its interactions with executive control (FP) network show frequency specific traits which differ in FA and OM meditation, with a crucial role played by the alpha rhythm. Moreover, coupling ranks might represent a marker of FA and OM meditation skills or expertise. Specifically, given that the posterior cingulate cortex (PCC) is the core node of the DMN (Buckner et al., 2008), in this work we assessed MEG functional connectivity by mapping phase-coherence with respect to the PCC node of the DMN across three experimental conditions: FA meditation, OM meditation and a rest condition. Our study involved a highly selected group of “virtuoso” meditators (Theravada Buddhist monks), with long-term training in both FA and OM meditation styles (see also Manna et al., 2010), emphasizing the cultivation of attention and awareness (monitoring) skills through all moments of their monastic life (Sumedho, 1994).

## Materials and methods

### Participants, procedures, and acquisition

An experienced meditator group of Theravada Buddhist monks was enrolled in this study. Specifically, the experienced meditator group consisted in eight Theravada Buddhist monks (all right handed males, mean age 37.9 years, range 25–53 years, SD 9.4 years), with, on average, over 15,750 h of meditation practice in Theravada Buddhist monasteries. The monks were recruited from the Santacittarama monastery, in Italy, where they follow a Thai Forest Tradition. In this tradition, monks experience regular intensive meditation retreats, with a balanced practice of *Samatha* (FA) and *Vipassana* (Open Monitoring, OM) meditation forms. These retreats also include a long winter retreat lasting for about 3 months. Outside the retreat periods, the monks typically practice 2 h per day balanced FA and OM meditation styles, with the monastery community. The experiment was conducted with the subject written informed consent according to the Declaration of Helsinki, as well as with the approval of the local responsible Ethical Committee. Only highly trained monk meditators were included in the study, with a minimal meditation expertise of about 2500 h, since an extensive training is necessary for reliably perform the two meditation styles. The same group underwent also fMRI scans which were analyzed in a previous study by our group (Manna et al., 2010).

**Figure 1:**
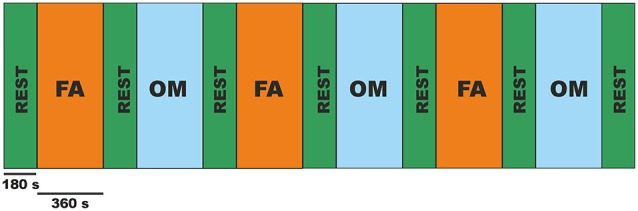
**Schematic representation of the block design paradigm alternating 6 min of focused attention meditation (FA) with 6 min of open monitoring meditation (OM) interleaved by 3 min of Resting State (REST)**.

The experimental paradigm consisted in a block design of 6 min FA meditation and 6 min OM meditation blocks, each preceded and followed by a 3 min non meditative resting state block (REST). Each sequence was repeated three times, see Figure 1. During all conditions, the subjects were sitting under the MEG scanner keeping their eyes closed and did not employ any discursive strategy, recitation, breath manipulation, or visualization technique.

**Figure 2:**
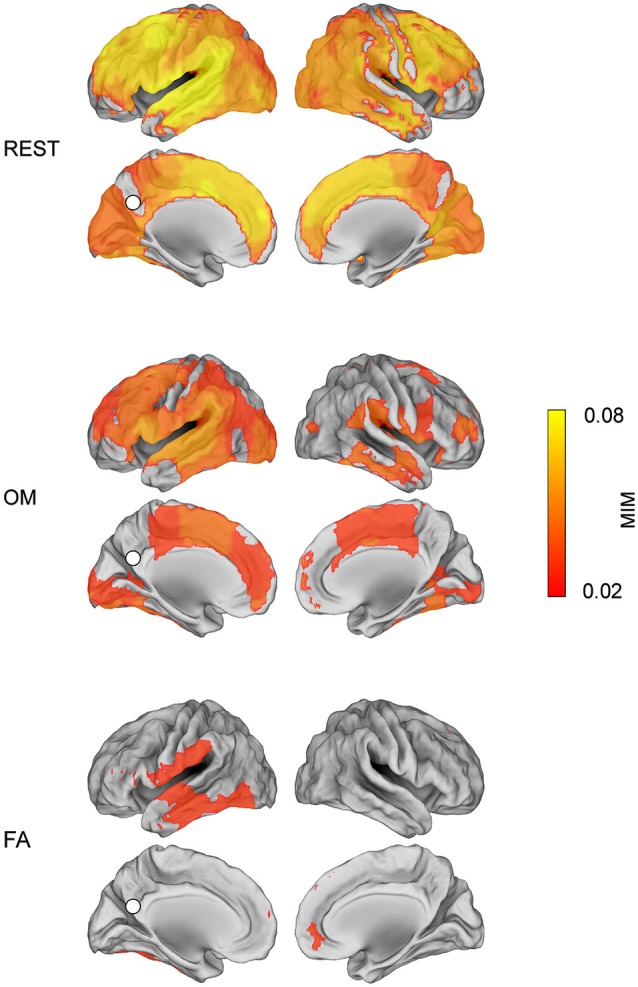
**Alpha band MIM connectivity map (*p* < 0.01, Bonferroni corrected value) with respect to the PCC seed in the three conditions: REST, OM meditation, FA meditation**.

The switch between conditions was instructed by the experimenter through an auditory word-signal consisting in the condition name, i.e., “Rest”, “Samatha”, “Vipassana” delivered via an interphone prior to the beginning of each recording block during a pause to the MEG acquisition between the end of one block and the beginning of the subsequent one (corresponding to the black vertical lines in the scheme of Figure 2).

At the end of the experiment, all participants were subject to a retrospective assessment of their ability to perform the task consisting in an in-house developed questionnaire. All participants reported they could correctly perform the three task conditions and experienced no differences in the difficulty in performing the two meditation styles. They also reported that the MEG scanner was perceived as a natural setting for mindfulness meditation thanks to the sitting position and the quiet environment.

MEG data were recorded by the 165-channel MEG system installed inside a magnetically shielded room at the Institute of Advanced Biomedical Technologies (ITAB), University of Chieti (Pizzella et al., 2001; Chella et al., 2012). This system includes 153 dc SQUID integrated magnetometers arranged on a helmet covering the whole head plus 12 reference channels. Electrocardiogram (ECG) and electro-oculogram (EOG) signals were also recorded for artifact rejection. All signals were band-pass filtered at 0.16–250 Hz and digitized at 1 kHz.

After each FA meditation, OM meditation and REST block, the position of the subject’s head with respect to the sensors was determined by acquiring the signal generated by five coils placed on the subject’s scalp before the starting of the MEG session. The coil positions, together with anatomical landmarks (left and right preauricular and nasion), were measured by means of a 3D digitizer (3Space Fastrak; Polhemus) allowing for the definition of a subject head based coordinate system and coregistration to magnetic resonance (MR) anatomical images. Magnetic resonance images were acquired using a sagittal magnetization prepared rapid acquisition gradient echo T1-weighted sequence (MP-RAGE; Siemens Vision scanner 1.5 T; TR = 9.7 s, echo time TE = 4 ms, alpha = 12°, inversion time = 1200 ms, voxel size = 1 × 1 × 1 mm^3^).

### MEG brain signal reconstruction

The recorded MEG data were pre-processed by using an independent components analysis based algorithm (Mantini et al., 2011). In brief, the algorithm projects the MEG data onto a set of maximally independent components and automatically classifies them, thus identifying artifactual components (e.g., cardiac artifact, eye movements) and components generated by brain signals. A similar classification procedure has also been employed in Saggar et al. (2012). Components classified of brain origin were thus projected at the brain level in order to identify the location and intensity of the corresponding neural source/sources. To this aim, the brain component topographies were input to a weighted Minimum-Norm Least Squares (wMNLS) linear inverse implemented in the Curry 6.0 (Neuroscan) analysis software (Fuchs et al., 1999). Brain currents were reconstructed on a Cartesian 3D grid with 4 mm step (i.e., 4 × 4 × 4 mm^3^ voxel size) bounded by the subject brain volume as derived from segmentation of individual MR images. Once the component topographies had been projected onto the brain space, the activity at each voxel at each sample in time was obtained as a linear combination of the component time courses weighted by their related brain source map. Further details are given in Marzetti et al. (2013).

### MEG functional connectivity

The estimated MEG brain signals were the starting point for the study of functional coupling of ongoing brain activity. Here, to map MEG functional connectivity we used an extension of the imaginary part of coherence for detecting lagged coupling, namely the Multivariate Interaction Measure (MIM; Ewald et al., 2012; Marzetti et al., 2013), that maximizes the imaginary part of coherence between a given reference voxel (*seed, s*) and any other voxel (*target, j*). More specifically, the estimated MEG signal at each brain voxel is a vector quantity that can be represented through its components in a given reference system. Multivariate Interaction Measure is designed to maximize the imaginary part of coherence between vector quantities. The mathematical details on MIM derivation can be found in Ewald et al. (2012). For the reader convenience, we briefly review MIM definition in the following.

Given the vector Fourier transformed signals as a function of frequency *f* at the seed and target voxels: *X_s_*(*f*) and *X_j_*(*f*), respectively, and introducing the compact notation *X*(*f*) = [$X_{s}^{T}$(*f*) $X_{j}^{T}$(*f*)], the cross-spectrum between the two vectors *X_s_*(*f*) and *X_j_*(*f*), can be written in the block form:

$$C(f)=\langle X(f)X(f)*\rangle =\left(\begin{array}{cc} C_{ss}^{R}(f)+JC_{ss}^{I}(f) & C_{sj}^{R}(f)+JC_{sj}^{I}(f)\\ C_{js}^{R}(f)+JC_{js}^{I}(f) & C_{jj}^{R}(f)+JC_{jj}^{I}(f) \end{array}\right)$$

and MIM between *s* and *j* is thus defined as:

$${\text{MIM}}_{\text{sj}}=tr\left({\left(C_{ss}^{R}\right)}^{-1}C_{sj}^{I}{\left(C_{jj}^{R}\right)}^{-1}{\left(C_{sj}^{I}\right)}^{T}\right)$$

In the above notation, *tr* indicates matrix trace, the T subscript indicates matrix transpose, superscripts R and I denote the real and the imaginary parts, the ^−1^ subscript indicates matrix inverse, the * subscript indicates matrix conjugate transpose, and the capital J indicates the imaginary unit. A more detailed recapitulation of the method is also given in Marzetti et al. (2013).

In this work, cross-spectra were estimated with Fast Fourier analysis after signal linear de-trending and Hanning windowing and were averaged using time epochs of 1.0 s duration with 50% overlap leading to a frequency resolution of 1 Hz. The number of averaged epochs is approximately 700 for each block of the OM and FA meditations and approximately 350 for each rest block.

The method, being based on the maximization of imaginary coherence, largely overcomes the well-known limitation to the study of functional connectivity by EEG/MEG posed by signal mixing artifacts, i.e., any active source in the brain contributes, in a weighted manner, to the signals measured at all sensors through volume spread (see Figure 2A in Engel et al., 2013). This effect constitutes an especially severe confound for estimates of brain interactions (Nolte et al., 2004; Marzetti et al., 2007; Schoffelen and Gross, 2009; Sekihara et al., 2011) and needs to be taken into account by mapping MEG functional connectivity through robust measures.

Functional connectivity through MIM was here estimated with a seed based approach, i.e., between the signal at the *seed* voxel and the signals at all other target brain voxels (approximately 28,000), and, in order to investigate the role of the DMN in frequency specific coupling to other brain networks in the different conditions, the seed was chosen in the PCC, the core node of the DMN (Buckner et al., 2008).

Functional connectivity was estimated for frequencies corresponding to the delta to gamma brain rhythms, i.e., from 2 to 80 Hz. To improve frequency specificity, consecutive frequency bins were further averaged over frequency bands defined on the basis of IAF peak. The alpha band was thus defined for each subject as IAF ± 2 Hz; the definitions of the other frequency bands were individually adjusted accordingly. On average, these bands span the following frequency ranges: delta (2–3.5 Hz), theta (4–7 Hz), alpha (8–12 Hz), beta (13–30 Hz), gamma (30–80 Hz) in accordance with conventional practice.

To investigate significant functional connectivity to PCC, a non-parametric Wilcoxon signed-rank test was used to assess voxel-wise significance across subjects (*p* < 0.01, Bonferroni corrected). For each frequency band, the MIM distribution across subjects for each voxel was compared to the empirical distribution of MIM for independent sources (simulated as independent and identically distributed, i.i.d., Gaussian noise) using a Monte Carlo approach with 20,000 repetitions. This procedure allowed to identify significant connections to PCC for each condition after Bonferroni correction for multiple comparisons.

A paired two tail *t*-test was used to compare MIM values between conditions (e.g., OM meditation condition and REST) and to derive t-contrast maps between condition pairs after false discovery rate correction (Benjamini and Hockberg, 1995) for multiple comparisons (*p* < 0.01, FDR corrected), thus highlighting brain regions that are differently involved in a specific meditative state.

Specifically, to the aim of understanding whether different meditation styles involve differential coupling between PCC and other brain areas in different frequency bands, we evaluated coupling to PCC in terms of t-maps for all possible contrast pairs: i.e., OM-FA, OM-REST, FA-REST. All maps were normalized to a common Montreal Neurological Institute (MNI) atlas through an affine transformation implemented in SPM8 (Friston, 2003) for comparison across subjects and projected to the standard brain surface for visualization by using the Caret software^1^ (Van Essen et al., 2001).

## Results

### State-dependent MEG functional connectivity

Figure 2 shows condition specific alpha band MIM maps of functional connectivity to PCC after correction for multiple comparisons, *p* < 0.01, Bonferroni corrected. The PCC seed is indicated with a white dot. Moreover, the areas whose MIM value is equal or larger than the 75% of the maximum MIM value for each condition are listed in Table 1. Specifically, in OM meditation this procedure identified the PCC coupling to left lateral temporal cortex (lLTC), left superior frontal gyrus (lSFG), left middle superior frontal gyrus (lMSFG), left anterior cingulate cortex (lACC), left dorsolateral prefrontal cortex (ldlPFC), left angular gyrus (lAG) and right ventral inferior temporal gyrus (rvITG). In FA meditation, this procedure identified the PCC coupling to rvITG, right and left lateral temporal cortices (LTC) and left inferior occipital lobe (lIOG). During REST, the highest coupling was observed with respect to left medial prefrontal cortex (lmPFC), lLTC, lSFG, lMSFG, lACC and lAG.

**Table 1:** **List of MNI coordinates for the areas most significantly connected to posterior cingulate cortex in the alpha band**.

	Hemisphere	*x* (mm)	*y* (mm)	*z* (mm)	ROI	
**OM**	L	−61	−33	−6	*LTC*
	L	−17	30	61	*SFG*
	L	−43	20	49	*MSFG*
	L	−7	0	50	*ACC*
	L	−23	31	48	*dlPFC*
	L	−64	−42	38	*AG*
	R	56	−41	14	*vITG*
**FA**	L	−26	−97	5	*IOG*
	L	−61	−11	−6	*LTC*
	R	56	−41	−14	*vITG*
	R	61	−22	−6	*LTC*
**REST**	L	−61	−33	−7	*LTC*
	L	−17	30	61	*SFG*
	L	−43	20	49	*MSFG*
	L	−7	0	50	*ACC*
	L	−64	−42	38	*AG*
	L	−4	46	12	*mPFC*
	R	4	0	47	*ACC*

*The Region of Interest (ROI) represent areas whose MIM value is equal or larger than the 75% of the maximum MIM value for each condition. The ROI labelling is as follows: lateral temporal cortex (LTC), superior frontal gyrus (SFG), middle superior frontal (MSFG), ventral inferior parietal sulcus (vIPS), anterior cingulate cortex (ACC), dorsolateral prefrontal cortex (dlPFC), angular gyrus (AG), ventral inferior temporal gyrus (vITG), inferior occipital gyrus (IOG), left medial prefrontal cortex (lmPFC)*.

**Figure 3:**
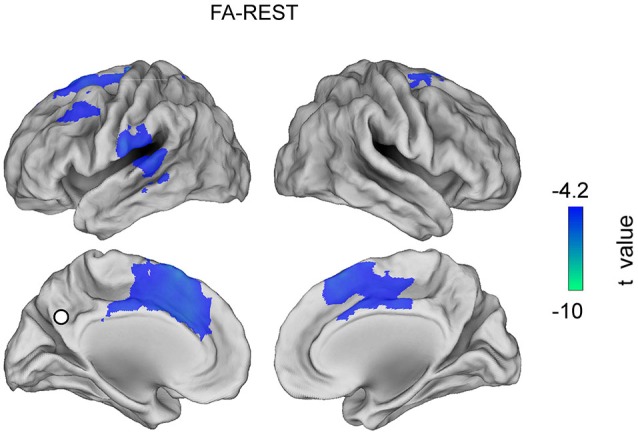
**T map for the contrast FA meditation vs. REST obtained in the alpha band (*p* < 0.01, FDR corrected value)**.

**Figure 4:**
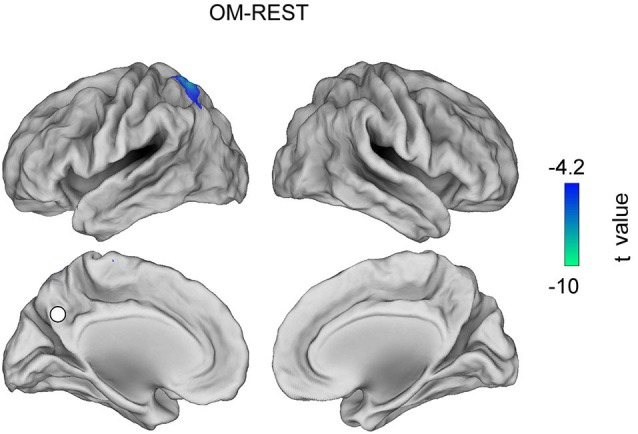
**T map for the contrast OM meditation vs. REST obtained in the alpha band (*p* < 0.01, FDR corrected value)**.

**Figure 5:**
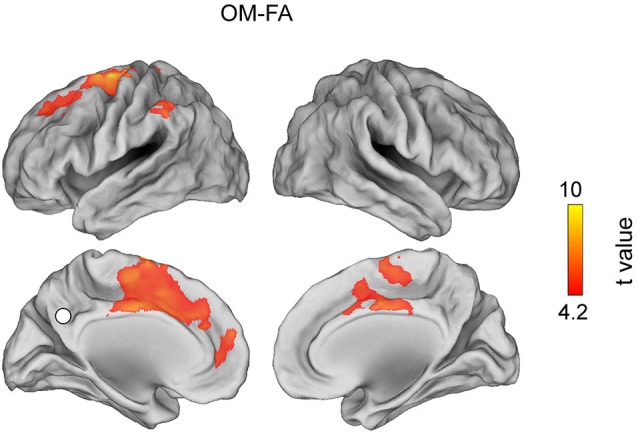
**T map for the contrast OM meditation vs. FA meditation in the alpha band (*p* < 0.01, corrected value)**.

Figures 3, 4, 5 show the topographies of significant t-maps (*p* < 0.01, FDR corrected) in alpha for all the contrast pairs, namely the FA meditation vs. REST contrast is shown in Figure 3; the OM meditation vs. REST contrast is shown in Figure 4 and the OM vs. FA meditation results are reported in Figure 5.

### Focused attention meditation vs. resting state

The alpha band connectivity in the contrast between FA meditation and REST (Figure 3) results in the PCC being more coupled to lSFG, left superior middle frontal gyrus (lSMFG), lLTC, left and right ACC during REST than during FA meditation. See Table 2.

**Table 2:** **List of ROIs significantly differently connected to posterior cingulate cortex in the alpha band in the contrast between conditions**.

	Hemisphere	*x* (mm)	*y* (mm)	*z* (mm)	ROI	Connectivity to PCC
FA-REST	L	−17	30	61	*SFG*	REST > FA
	L	−43	18	43	*MSFG*	REST > FA
	L	−61	−33	−6	*LTC*	REST > FA
	L	−7	0	50	*ACC*	REST > FA
	R	21	8	69	*SFG*	REST > FA
	R	10	0	51	*ACC*	REST > FA
OM-REST	L	−22	−58	67	*IPS*	REST > OM
OM-FA	L	−4	46	12	*mPFC*	OM > FA
	L	−28	−4	69	*SFG*	OM > FA
	L	−23	31	48	*dlPFC*	OM > FA
	L	−1	−2	44	*ACC*	OM > FA
	L	−58	−37	42	*IPL*	OM > FA
	R	2	0	46	*ACC*	OM > FA

### Open monitoring meditation vs. resting state

The contrast between OM meditation and REST results in only the left intraparietal sulcus being significantly less connected to PCC during OM meditation than during REST.

### Open monitoring vs. focused attention meditation

The contrast between OM meditation and FA meditation (Figure 5) highlights the stronger alpha band coupling during OM meditation of PCC with the following regions: lmPFC, lSFG, lSMFG, ldlPFC and lACC, left inferior parietal lobule (lIPL). See Table 2.

### Frequency specificity

**Figure 6:**
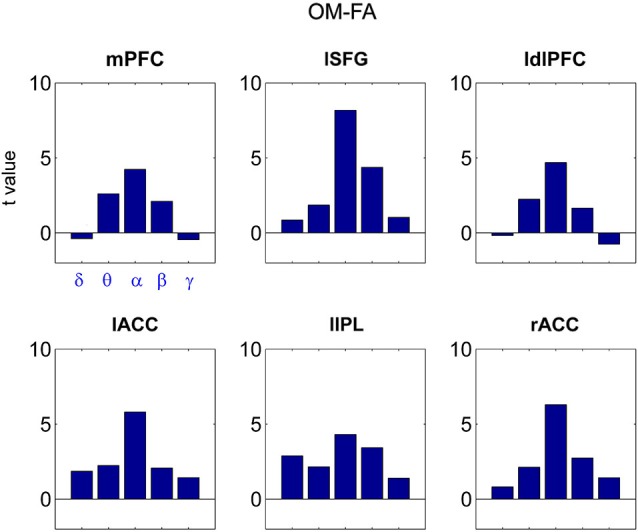
**Bar graphs of t values for the mPFC, lSFG, ldlPFC, lACC, lIPL and rACC regions emerged from the contrast OM vs. FA across the delta (δ), theta (θ), alpha (α), beta (β) and gamma (γ) frequency bands**.

Notably, the coupling of PCC to the nodes of Default Mode and Fronto Parietal networks as listed in Table 2 was a specific signature of the alpha band as it was not observed in the other frequency bands included in the analysis. Figure 6 shows the t values, represented as bar graphs, for the OM vs. FA contrast. T values were extracted for the ROI coordinates listed in Table 2 for OM-FA for the delta (δ), theta (θ), alpha (α), beta (β) and gamma (γ) frequency bands. This is a representative situation showing the frequency specificity of the observed coupling.

### Correlation with meditation expertise

**Figure 7:**
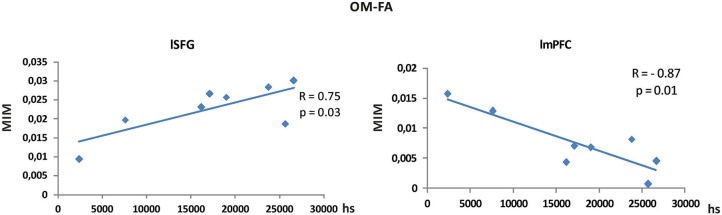
**Pearson correlations between MIM connectivity differences in OM vs. FA meditations and expertise expressed in meditation hours**. Left: correlation value between MIM differences in lSFG is significantly (*p* = 0.03) found to be *R* = 0.75, Right: correlation value between MIM differences in lmPFC is significantly (*p* = 0.01) found to be *R* = −0.87.

To evaluate to what extent the alpha band differences between conditions reported in Figures 3, 4, 5 might be explained by differences in meditation expertise within the monk group, we correlated the difference between MIM values in the two conditions with meditation expertise measured as overall meditation hours, for all brain areas listed in Table 2. Significant Pearson correlation was found in the contrast between OM and FA meditation for the lSFG and lmPFC nodes. The difference was calculated using values extracted from the voxel closest (according to Euclidean distance) to the MNI coordinate listed in Table 2. Nevertheless, consistent results were observed in the node surroundings within a sphere of about 1 cm diameter. Specifically, a positive correlation (*R* = 0.75, *p* = 0.03) was found for the difference of MIM values between OM and FA meditations in lSFG and a negative correlation (*R* = −0.87, *p* = 0.01) was found in lmPFC. See Figure 7. All the other brain regions and condition contrasts did not show a significant correlation with meditation expertise.

## Discussion

This study aimed at investigating the effects of FA and OM meditation on MEG functional connectivity to the PCC, a crucial DMN node (Raichle et al., 2001; Buckner et al., 2008), in skilled long-term FA/OM meditators. Overall, the results indicate that the different conditions modulate the coupling between PCC and nodes of the DMN and of the FP (executive) network (Vincent et al., 2008) in the alpha band. The temporal richness of the MEG signal allowed us to quantify functional connectivity by frequency specific patterns able to capture stationary properties of network interactions in the brain. This goal was here achieved in a way robust to possible self-coupling confounds deriving by MEG poor spatial resolution and signal mixing artifacts.

Our phase coherence based approach was able to reveal stable functional connectivity patterns with respect to PCC across the meditation conditions and during the resting state. As shown in Figure 2, an overall reduced connectivity was observed during the two meditation conditions in comparison to the resting state, consistently with the EEG findings reported in Lehmann et al. (2012) and Berkovich-Ohana et al. (2013). This reduced connectivity was here expressed both in terms of lower average MIM values and of more localized connectivity topographies in meditation. Moreover, high topographical similarity was shared between the resting state and the OM meditation condition, compared to all other condition pairs.

When FA meditation was compared to the resting state, see Figure 3, a lower engagement of left and right superior frontal gyrus, lMSFG and lateral temporal cortex and of left and right anterior cingulate cortex was observed. Indeed, in line with an earlier fMRI study (Manna et al., 2010), functional deactivations during FA meditation as compared to the resting state involve executive (FP) areas, which have been found to activate with the DMN during mind wandering in another study (Christoff et al., 2009).

When OM meditation was compared to the resting state, see Figure 4, the left intraparietal sulcus was more connected to PCC during REST than during OM meditation. The left intraparietal sulcus may thus be more involved in executive and monitoring functions in OM meditation (Szameitat et al., 2002; Lutz et al., 2008), rather than being as coupled to the DMN (PCC) as in the resting state.

More interestingly, when OM meditation was directly compared to FA meditation, see Figure 5, regions in the left hemisphere belonging to the DMN (i.e., lmPFC) and the FP network (i.e., lACC, ldlPFC and left inferior parietal lobe) were more functionally connected to PCC. Also lSFG, an area which has been involved in both self-referential processing, linked to the DMN (e.g., Goldberg et al., 2006), and executive functions and monitoring (e.g., du Boisgueheneuc et al., 2006), were more functionally connected to PCC during OM meditation in comparison to FA meditation. The higher coupling within the DMN can be related to the higher occurrence of thoughts and mental images during OM meditation than during FA meditation (Lutz et al., 2008; Raffone and Srinivasan, 2009). Similarly, the coupling with the lSFG can be related to an increased meta-awareness or monitoring of ongoing thoughts and mental images generated with involvement of the DMN, in OM as compared to FA meditation (Lutz et al., 2008; Raffone and Srinivasan, 2009). Indeed, the increased monitoring (mindfulness) during OM meditation would lead to a meta-awareness that enables the maintenance of the meditative state even in presence of spontaneous mentation. This mindful observing ability is plausibly more developed in meditators with a higher level of expertise (Figure 7; Lutz et al., 2008), in line with our results here for (positive) correlation between functional connectivity to PCC and meditation expertise, showing that coupling is differentially regulated by meditation expertise in lSFG. Finally, a negative correlation between functional coupling of lmPFC to PCC and meditation expertise was found, as related to the OM vs. FA meditation contrast, which appears consistent with an expected reduced identification (self-reference) of more expert meditators with ongoing mental activity in any of the two forms of meditation (Lutz et al., 2008). This evidence may also be related to an attenuated medial prefrontal cortex activation with emotional thought contents (Northoff et al., 2004), likely to arise with OM meditation as compared to FA meditation, with meditation expertise. By contrast, meditators with a lower expertise exhibited a higher modulation of the coupling between PCC and lmPFC within the DMN in shifting between FA (with lower coupling) and OM (with higher coupling) meditation styles.

As shown in earlier neuroimaging studies, the lSFG is involved in self-related processing and awareness (Goldberg et al., 2006), which can be related to the notion of “narrative self” (Gazzaniga, 1995). In this respect, based on neuroimaging results, theorists have suggested that the brain systems for the reflective self are likely to involve lateral prefrontal areas, and not just midline DMN areas (Northoff et al., 2006; Tagini and Raffone, 2010). Our present study further suggests that the neural mechanisms for the “narrative self” may involve the crucial coupling between PCC and lSFG in the alpha band, which can be modulated by meditation, with differential effects of FA and OM styles. Since the lSFG has also been related to executive and monitoring functions (e.g., in working memory) (du Boisgueheneuc et al., 2006), the differential coupling between PCC and lSFG in the alpha band found in our study may be linked to a differential recruitment of neuronal populations in the lSFG for self-referential thought vs. executive and cognitive monitoring. Indeed, neuronal responses in lateral prefrontal cortex are highly adaptive, depending on the task setting and individual differences (Duncan, 2001). Interestingly, as suggested by our results, different styles of meditation and meditation expertise appeared to modulate such coupling and possibly the dynamic balance between the recruitment of FP and DMN networks.

Notably, all of the observed effects were specific to the alpha band (Figure 6). Besides the idling hypothesis, the role of alpha band phase coupling in meditation might be closely related to the role of alpha band synchronization as a functional mechanism of attention and consciousness (Palva and Palva, 2007; Knyazev, 2013). Indeed, an inhibitory role has been associated to the alpha rhythm to the aim of filtering out irrelevant sensory inputs (Klimesch et al., 2007; Bonnefond and Jensen, 2013) in a broad range of information processing tasks including selective spatial attention (Rihs et al., 2007; Foxe and Snyder, 2011; Van Ede et al., 2011) and working memory (Sauseng et al., 2009; Hagens et al., 2010; Spitzer and Blankenburg, 2011; Bonnefond and Jensen, 2012). Evidence indeed suggests that FA and OM meditation styles entail unique sets of attention and consciousness, and are not merely degrees of a state of relaxation (Dunn et al., 1999). More specifically, our findings highlighted a possible role of such rhythm in maintaining the stability of DMN internal phase coupling and subserving its modulation with the FP network, according to the specific meditation state. Indeed, the presently observed findings are consistent with a top-down alpha modulation hypothesis as a mechanism involved in stress-therapy meditation (Kerr et al., 2011), and with the idea that cooperation between the DMN and the FP network helps sustain monitoring of thoughts against compulsory self-reference (identification) (Farb et al., 2007) and interference (Smallwood et al., 2012). Taken together, our results support the idea that an interplay between the DMN and the FP network is crucial for the transition from resting state to different meditative states.

It has to be noted that it is not trivial that nodes classically ascribed by fMRI as belonging to the DMN and the FP should emerge as coupled to PCC also in MEG connectivity studies. Indeed, MEG provides a window into the high complexity of brain information processing at the temporal scales relevant for behavior which translate into frequency resolved coupling. For this reason, different systems comprised by some of the network nodes classically identified by fMRI might be represented by MEG phase coherence at different frequency scales, possibly speaking for a functional dissociation of network subsystems in the frequency domain.

A possible limitation of this study is the relatively small number of subjects included in the analysis. Our emphasis was indeed on the high (“virtuoso”) skills in both FA and OM meditation of the Theravada Buddhist monks involved in our study, appearing as a rare and selected sample in literature. Moreover, we did not include a comparison with a group of novice practitioners. In this respect, however, a recent fMRI study (Manna et al., 2010) with the same participants performing FA and OM meditation, found the most relevant differences in the contrasts between FA meditation, OM meditation and resting state conditions within the monk group, in line with our present focus. Indeed, for novice meditators it might be difficult to control for and to differentiate between the meditative states which is the primary focus of this study. As a general remark, it is indeed difficult to objectively validate what the participants are doing in a task which involves inherently subjective and covert states, such as FA and OM meditations. We believe that our involvement of meditators (Theravada Buddhist monks) who are expert in both FA and OM meditation has minimized the chances of inaccurate performance of the two meditation tasks, and this was also evident from the retrospective reports. Finally, it would be worth in the future to include in the protocol a controlled manipulation of attention by introducing a task (e.g., a sustained response inhibition task as in Zanesco et al., 2013). This would allow to e.g., compare task based functional connectivity before and after the meditation blocks and to correlate connectivity results with task performance.

## Conclusions

To summarize, the present study allowed to characterize the coupling of the major DMN node, the PCC, with the rest of the brain, and highlighted, in a data driven manner, its coupling to nodes of DMN and FP network specific to the alpha band. Our findings showed that the alpha band is selectively involved in the different couplings of PCC in the two meditation styles and during rest, and that a stable coupling within DMN and between DMN and FP network characterized the contrast between the two meditation styles, which was correlated to meditation expertise.

More generally, MEG functional connectivity was able to reveal important features of meditative states in the brain which were modulated by expertise. Indeed, MEG can provide a unique neuroimaging tool to study meditation and mindfulness processes thanks to its ability to recover brain functional coupling in a frequency resolved manner, thus reconciling the long tradition of the EEG based approach with an fMRI network based approach to meditation. Not secondary to this aim, the MEG scanner appeared as a more convenient setting for performing research on meditation thanks to the sitting position that the subject can maintain during the measurement, as well as to the absence of any disturbing external noise inside the shielded room in which the MEG system is set.

## Conflict of interest statement

The authors declare that the research was conducted in the absence of any commercial or financial relationships that could be construed as a potential conflict of interest.

## Footnotes

^1^http://www.nitrc.org/projects/caret/

## References

[B1] Benjamini Y. Hockberg Y. (1995). Controlling the false discovery rate: a practical and powerful approach to multiple testing. J. R. Statist. Soc. B 57, 289–300

[B2] Berger H. I. (1929). Über das Elektroenkephalogram des Menschen. Arch. Psychiat. Nervenkr. 87, 527–570 10.1007/BF01797193

[B3] Berkovich-Ohana A. Glicksohn J. Goldstein A. (2013). Studying the default mode and its mindfulness-induced changes using EEG functional connectivity. Soc. Cogn. Affect. Neurosci. [Epub ahead of print]. 10.1093/scan/nst153 24194576 PMC4187278

[B4] Bonnefond M. Jensen O. (2012). Alpha oscillations serve to protect working memory maintenance against anticipated distracters. Curr. Biol. 22, 1969–1974 10.1016/j.cub.2012.08.029 23041197

[B5] Bonnefond M. Jensen O. (2013). The role of gamma and alpha oscillations for blocking out distraction. Commun. Integr. Biol. 6:e22702 10.4161/cib.22702 23802042 PMC3689574

[B6] Brewer J. A. Worhunsky P. D. Gray J. R. Tang Y. Weber J. Kober H. (2011). Meditation experience is associated with differences in default mode network activity and connectivity. Proc. Natl. Acad. Sci. U S A 108, 20254–20259 10.1073/pnas.1112029108 22114193 PMC3250176

[B7] Buckner R. L. Andrews-Hanna J. R. Schacter D. L. (2008). The brain’s default network: anatomy, function and relevance to disease. Ann. N Y Acad. Sci. 1124, 1–38 10.1196/annals.1440.011 18400922

[B8] Cahn B. R. Polich J. (2006). Meditation states and traits: EEG, ERP and neuroimaging studies. Psychol. Bull. 132, 180–211 10.1037/0033-2909.132.2.180 16536641

[B9] Chella F. Zappasodi F. Marzetti L. Della Penna S. Pizzella V. (2012). Calibration of a multichannel meg system based on the signal space separation method. Phys. Med. Biol. 57, 4855–4870 10.1088/0031-9155/57/15/4855 22797687

[B10] Chiesa A. Serretti A. (2010). A systematic review of neurobiological and clinical features of mindfulness meditations. Psychol. Med. 40, 1239–1252 10.1017/S0033291709991747 19941676

[B11] Christoff K. Gordon A. M. Smallwood J. Smith R. Schooler J. W. (2009). Experience sampling during fMRI reveals default network and executive system contributions to mind wandering. Proc. Natl. Acad. Sci. U S A 106, 8719–8724 10.1073/pnas.0900234106 19433790 PMC2689035

[B12] Cooper N. R. Croft R. J. Dominey S. J. J. Burgess A. P. Gruzelier J. H. (2003). Paradox lost? Exploring the role of alpha oscillations during externally vs. internally directed attention and the implications for idling and inhibition hypotheses. Int. J. Psychophysiol. 47, 65–74 10.1016/s0167-8760(02)00107-1 12543447

[B13] de Pasquale F. Della Penna S. Snyder A. Z. Marzetti L. Pizzella V. Romani G. L. (2012). A cortical core for dynamic integration of functional networks in the resting human brain. Neuron 74, 753–764 10.1016/j.neuron.2012.03.031 22632732 PMC3361697

[B14] de Pasquale F. Marzetti L. (2014). “Temporal and spectral signatures of the default mode network,” in MEG: From Signal to Dynamic Cortical Networks, eds Supek S. Aine C. J. (Heidelberg: Springer Verlang), 451–476

[B15] du Boisgueheneuc F. Levy R. Volle E. Seassau M. Duffau H. Kinkingnehun S. (2006). Functions of the left superior frontal gyrus in humans: a lesion study. Brain 129, 3315–3328 10.1093/brain/awl244 16984899

[B16] Duncan J. (2001). An adaptive coding model of neural function in prefrontal cortex. Nat. Rev. Neurosci. 2, 820–829 10.1038/35097575 11715058

[B17] Dunn B. R. Hartigan J. A. Mikulas W. L. (1999). Concentration and mindfulness meditations: unique forms of consciousness? Appl. Psychophysiol. Biofeedback 24, 147–165 10.1023/A:1023498629385 10652635

[B18] Engel A. K. Gerloff C. Hilgetag C. C. Nolte G. (2013). Intrinsic coupling modes: multiscale interactions in ongoing brain activity. Neuron 80, 867–886 10.1016/j.neuron.2013.09.038 24267648

[B19] Ewald A. Marzetti L. Zappasodi F. Meinecke F. C. Nolte G. (2012). Estimating true brain connectivity from EEG/MEG data invariant to linear and static transformations in sensor space. Neuroimage 60, 476–488 10.1016/j.neuroimage.2011.11.084 22178298

[B20] Farb N. A. Segal Z. V. Mayberg H. Bean J. McKeon D. Fatima Z. (2007). Attending to the present: mindfulness meditation reveals distinct neural modes of self-reference. Soc. Cogn. Affect. Neurosci. 2, 313–322 10.1093/scan/nsm030 18985137 PMC2566754

[B21] Foxe J. J. Snyder A. C. (2011). The role of alpha-band brain oscillations as a sensory suppression mechanism during selective attention. Front. Psychol. 2:154 10.3389/fpsyg.2011.00154 21779269 PMC3132683

[B22] Fries P. (2005). A mechanism for cognitive dynamics: neural communication through neuronal coherence. Trends Cogn. Sci. 9, 474–480 10.1016/j.tics.2005.08.011 16150631

[B23] Friston K. (2003). “Introduction: experimental design and statistical parametric mapping,” in Human Brain Function. 2nd Edn., eds Frackowiak R. Friston K. Frith C. Dolan R. Price C. (San Diego: Academic Press), 599–634

[B24] Fuchs M. Wagner M. Köhler T. Wischmann H. A. (1999). Linear and nonlinear current density reconstructions. J. Clin. Neurophysiol. 16, 267–295 10.1097/00004691-199905000-00006 10426408

[B25] Gazzaniga M. S. (1995). Principles of human brain organization derived from split-brain studies. Neuron 14, 217–228 10.1016/0896-6273(95)90280-5 7857634

[B26] Goldberg I. I. Harel M. Malach R. (2006). When the brain loses its self: prefrontal inactivation during sensorimotor processing. Neuron 50, 329–339 10.1016/j.neuron.2006.03.015 16630842

[B27] Goldstein J. Kornfield J. (2001). Seeking the Heart of Wisdom: The Path of Insight Meditation. Boston, MA: Shambhala

[B28] Hagens S. Osipova D. Oostenveld R. Jensen O. (2010). Somatosensory working memory performance in humans depends on both engagement and disengagement of regions in a distributed network. Hum. Brain Mapp. 31, 26–35 10.1002/hbm.20842 19569072 PMC6871021

[B29] Hari R. Salmelin R. Mäkelä J. P. Salenius S. Helle M. (1997). Magnetoencephalographic cortical rhythms. Int. J. Psychophysiol. 26, 51–62 10.1016/s0167-8760(97)00755-1 9202994

[B30] Hasenkamp W. Wilson-Mendenhall C. D. Duncan E. Barsalou L. W. (2012). Mind wandering and attention during focused meditation: a fine-grained temporal analysis of fluctuating cognitive states. Neuroimage 59, 750–760 10.1016/j.neuroimage.2011.07.008 21782031

[B31] Ivanovski B. Malhi G. S. (2007). The psychological and neurophysiological concomitants of mindfulness forms of meditation. Acta Neuropsychiatr. 19, 76–91 10.1111/j.1601-5215.2007.00175.x 26952819

[B32] Jann K. Dierks T. Boesch C. Kottlow M. Strik W. Koenig T. (2009). BOLD correlates of EEG alpha phase-locking and the fMRI default mode network. Neuroimage 45, 903–916 10.1016/j.neuroimage.2009.01.001 19280706

[B33] Kabat-Zinn J. (1994). Wherever You Go, There You Are: Mindfulness Meditation in Everyday Life. New York, NY: Hyperion

[B34] Keng S. L. Smoski M. J. Robins C. J. (2011). Effects of mindfulness on psychological health: a review of empirical studies. Clin. Psychol. Rev. 31, 1041–1056 10.1016/j.cpr.2011.04.006 21802619 PMC3679190

[B35] Kerr C. E. Jones S. R. Wan Q. Pritchett D. L. Wasserman R. H. Wexler A. (2011). Effects of mindfulness meditation training on anticipatory alpha modulation in primary somatosensory cortex. Brain Res. Bull. 85, 96–103 10.1016/j.brainresbull.2011.03.026 21501665

[B36] Killingsworth M. A. Gilbert D. T. (2010). A wandering mind is an unhappy mind. Science 330:932 10.1126/science.1192439 21071660

[B38] Klimesch W. (1996). Memory processes, brain oscillations and EEG synchronization. Int. J. Psychophysiol. 24, 61–100 10.1016/s0167-8760(96)00057-8 8978436

[B37] Klimesch W. (1999). EEG alpha and theta oscillations reflect cognitive and memory performance: a review and analysis. Brain Res. Brain Res. Rev. 29, 169–195 10.1016/s0165-0173(98)00056-3 10209231

[B39] Klimesch W. Sauseng P. Hanslmayr S. (2007). EEG alpha oscillations: the inhibition-timing hypothesis. Brain Res. Rev. 53, 63–88 10.1016/j.brainresrev.2006.06.003 16887192

[B40] Knyazev G. G. (2013). EEG correlates of self-referential processing. Front. Hum. Neurosci. 7:264 10.3389/fnhum.2013.00264 23761757 PMC3674309

[B41] Knyazev G. G. Slobodskoj-Plusnin J. Y. Bocharov A. V. Pylkova L. V. (2011). The default mode network and EEG alpha oscillations: an independent component analysis. Brain Res. 1402, 67–79 10.1016/j.brainres.2011.05.052 21683942

[B42] Kounios J. Beeman M. (2009). The Aha! moment. The cognitive neuroscience of insight. Curr. Dir. in Psycho. Sci. 18, 210–216 10.1111/j.1467-8721.2009.01638.x

[B43] Lachaux J. P. Rodriguez E. Martinerie J. Varela F. (1999). Measuring phase synchrony in the brain. Hum. Brain Mapp. 8, 194–208 10.1002/(sici)1097-0193(1999)8:4<194::aid-hbm4>3.0.co;2-c 10619414 PMC6873296

[B44] Larson-Prior L. J. Oostenveld R. Della Penna S. Michalareas G. Prior F. Babajani-Feremi A. (2013). Adding dynamics to the human connectome project with MEG. Neuroimage 80, 190–201 10.1016/j.neuroimage.2013.05.056 23702419 PMC3784249

[B45] Laufs H. Kleinschmidt A. Beyerle A. Eger E. Salek-Haddadi A. Preibisch C. (2003a). EEG-correlated fMRI of human alpha activity. Neuroimage 19, 1463–1476 10.1016/s1053-8119(03)00286-6 12948703

[B46] Laufs H. Krakow K. Sterzer P. Eger E. Beyerle A. Salek-Haddadi A. (2003b). Electroencephalographic signatures of attentional and cognitive default modes in spontaneous brain activity at rest. Proc. Natl. Acad. Sci. U S A 100, 11053–11058 10.1073/pnas.1831638100 12958209 PMC196925

[B47] Lehmann D. Faber P. L. Tei S. Pascual-Marqui R. D. Milz P. Kochi K. (2012). Reduced functional connectivity between cortical sources in five meditation traditions detected with lagged coherence using EEG tomography. Neuroimage 60, 1574–1586 10.1016/j.neuroimage.2012.01.042 22266174

[B48] Lutz A. Slagter H. A. Dunne J. D. Davidson R. J. (2008). Attention regulation and monitoring in meditation. Trends Cogn. Sci. 12, 163–169 10.1016/j.tics.2008.01.005 18329323 PMC2693206

[B49] Malinowski P. (2013a). Neural mechanisms of attentional control in mindfulness meditation. Front. Neurosci. 7:8 10.3389/fnins.2013.00008 23382709 PMC3563089

[B50] Malinowski P. (2013b). “Flourishing through meditation and mindfulness”, in Oxford Hand book of Happiness, eds David S. Boniwell I. Conley Ayers A. (Oxford: Oxford University Press), 384–396

[B51] Manna A. Raffone A. Perrucci M. G. Nardo D. Ferretti A. Tartaro A. (2010). Neural correlates of focused attention and cognitive monitoring in meditation. Brain Res. Bull. 82, 46–56 10.1016/j.brainresbull.2010.03.001 20223285

[B52] Mantini D. Della Penna S. Marzetti L. de Pasquale F. Pizzella V. Corbetta M. (2011). A signal processing pipeline for Magnetoencephalography resting state networks. Brain Connect. 1, 49–59 10.1089/brain.2011.0001 22432954

[B53] Mantini D. Perrucci M. G. Del Gratta C. Romani G. L. Corbetta M. (2007). Electrophysiological signatures of resting state networks in the human brain. Proc. Natl. Acad. Sci. U S A 104, 13170–13175 10.1073/pnas.0700668104 17670949 PMC1941820

[B54] Marzetti L. Della Penna S. Snyder A. Z. Pizzella V. Nolte G. de Pasquale F. (2013). Frequency specific interactions of MEG resting state activity within and across brain networks as revealed by the multivariate interaction measure. Neuroimage 79, 172–183 10.1016/j.neuroimage.2013.04.062 23631996 PMC3843123

[B55] Marzetti L. Nolte G. Perrucci M. G. Romani G. L. Del Gratta C. (2007). The use of standardized infinity reference in EEG coherency studies. Neuroimage 36, 48–63 10.1016/j.neuroimage.2007.02.034 17418592

[B56] Michels L. Bucher K. Lüchinger R. Klaver P. Martin E. Jeanmonod D. (2010). Simultaneous EEG-fMRI during a working memory task: modulations in low and high frequency bands. PLoS One 5:e10298 10.1371/journal.pone.0010298 20421978 PMC2858659

[B57] Nolfe G. (2011). EEG and meditation. Clin. Neurophysiol. 123, 631–632 10.1016/j.clinph.2011.08.016 21924677

[B58] Nolte G. Bai O. Wheaton L. Mari Z. Vorbach S. Hallet M. (2004). Identifying true brain interaction from EEG data using the imaginary part of coherency. Clin. Neurophysiol. 115, 2292–2307 10.1016/j.clinph.2004.04.029 15351371

[B59] Northoff G. Heinzel A. Bermpohl F. Niese R. Pfennig A. Pascual-Leone A. (2004). Reciprocal modulation and attenuation in the prefrontal cortex: an fmri study on emotional-cognitive interaction. Hum. Brain Mapp. 21, 202–212 10.1002/hbm.20002 14755839 PMC6871994

[B60] Northoff G. Heinzel A. de Greck M. Bermpohl F. Dobrowolny H. Panksepp J. (2006). Self-referential processing in our brain—a meta-analysis of imaging studies on the self. Neuroimage 31, 440–457 10.1016/j.neuroimage.2005.12.002 16466680

[B61] Ottaviani C. Shapiro D. Couyoumdjian A. (2013). Flexibility as the key for somatic health: from mind wandering to perseverative cognition. Biol. Psychol. 94, 38–43 10.1016/j.biopsycho.2013.05.003 23680439

[B62] Palva S. Palva J. M. (2007). New vistas for a-frequency band oscillations. Trends Neurosci. 30, 150–158 10.1016/j.tins.2007.02.001 17307258

[B63] Palva J. M. Palva S. Kaila K. (2005). Phase synchrony among neuronal oscillations in the human cortex. J. Neurosci. 25, 3962–3972 10.1523/jneurosci.4250-04.2005 15829648 PMC6724920

[B64] Pfurtscheller G. (2003). Induced oscillations in the alpha band: functional meaning. Epilepsia 44, 2–8 10.1111/j.0013-9580.2003.12001.x 14641556

[B65] Pizzella V. Della Penna S. Del Gratta C. Romani G. L. (2001). SQUID systems for biomagnetic imaging. Supercond. Sci. Technol. 14, R79–R114 10.1088/0953-2048/14/7/201

[B66] Raffone A. Srinivasan N. (2009). An adaptive workspace hypothesis about the neural correlates of consciousness: insights from neuroscience and meditation studies. Prog. Brain Res. 176, 161–180 10.1016/S0079-6123(09)17620-3 19733756

[B67] Raffone A. Srinivasan N. (2010). The exploration of meditation in the neuroscience of attention and consciousness. Cogn. Process. 11, 1–7 10.1007/s10339-009-0354-z 20041276

[B68] Raichle M. MacLeod A. M. Snyder A. Z. Powers W. J. Gusnard D. A. Shulman G. L. (2001). The default mode of brain function. Proc. Natl. Acad. Sci. U S A 98, 676–682 10.1073/pnas.98.2.676 11209064 PMC14647

[B69] Rihs T. A. Michel C. M. Thut G. (2007). Mechanisms of selective inhibition in visual spatial attention are indexed by alpha-band EEG synchronization. Eur. J. Neurosci. 25, 603–610 10.1111/j.1460-9568.2007.05278.x 17284203

[B71] Sadaghiani S. Scheeringa R. Lehongre K. Morillon B. Giraud A. L. D’ Esposito M. (2012). *α*-band phase synchrony is related to activity in the fronto-parietal adaptive control network. J. Neurosci. 32, 14305–14310 10.1523/JNEUROSCI.1358-12.2012 23055501 PMC4057938

[B70] Sadaghiani S. Scheeringa R. Lehongre K. Morillon B. Giraud A. L. Kleinschmidt A. (2010). Intrinsic connectivity networks, alpha oscillations and tonic alertness: a simultaneous electroencephalography/functional magnetic resonance imaging study. J. Neurosci. 30, 10243–10250 10.1523/JNEUROSCI.1004-10.2010 20668207 PMC6633365

[B72] Saggar M. King B. G. Zanesco A. P. Maclean K. A. Aichele S. R. Jacobs T. L. (2012). Intensive training induces longitudinal changes in meditation state-related EEG oscillatory activity. Front. Hum. Neurosci. 6:256 10.3389/fnhum.2012.00256 22973218 PMC3437523

[B73] Sauseng P. Klimesch W. Heise K. F. Gruber W. R. Holz E. Karim A. A. (2009). Brain oscillatory substrates of visual short-term memory capacity. Curr. Biol. 19, 1846–1852 10.1016/j.cub.2009.08.062 19913428

[B74] Schoffelen J. M. Gross J. (2009). Source connectivity analysis with MEG and EEG. Hum. Brain Mapp. 30, 1857–1865 10.1002/hbm.20745 19235884 PMC6870611

[B75] Sekihara K. Owen J. P. Trisno S. Nagarajan S. S. (2011). Removal of spurious coherence in MEG source-space coherence analysis. IEEE Trans. Biomed. Eng. 58, 3121–3129 10.1109/TBME.2011.2162514 21824842 PMC4096348

[B76] Smallwood J. Brown K. Baird B. Schooler J. W. (2012). Cooperation between the default mode network and the frontal-parietal network in the production of an internal train of thought. Brain Res. 1428, 60–70 10.1016/j.brainres.2011.03.072 21466793

[B77] Spitzer B. Blankenburg F. (2011). Stimulus-dependent EEG activity reflects internal updating of tactile working memory in humans. Proc. Natl. Acad. Sci. U S A 108, 8444–8449 10.1073/pnas.1104189108 21536865 PMC3100957

[B78] Spreng R. N. Schacter D. L. (2012). Default network modulation and large-scale network interactivity in healthy young and old adults. Cereb. Cortex 22, 2610–2621 10.1093/cercor/bhr339 22128194 PMC3464415

[B79] Spreng R. N. Stevens W. D. Chamberlain J. P. Gilmore A. W. Schacter D. L. (2010). Default network activity, coupled with the frontoparietal control network, supports goal-directed cognition. Neuroimage 53, 303–317 10.1016/j.neuroimage.2010.06.016 20600998 PMC2914129

[B80] Sumedho A. (1994). The Mind and the Way: Buddhist Reflections on Life. Somerville, MA: Wisdom Publications

[B81] Szameitat A. J. Schubert T. Müller K. Von Cramon D. Y. (2002). Localization of executive functions in dual-task performance with fMRI. J. Cogn. Neurosci. 14, 1184–1199 10.1162/089892902760807195 12495525

[B82] Tagini A. Raffone A. (2010). The ‘I’ and the ‘Me’ in self-referential awareness: a neurocognitive hypothesis. Cogn. Process. 11, 9–20 10.1007/s10339-009-0336-1 19763648

[B83] Tang Y. Y. Rothbart M. K. Posner M. I. (2012). Neural correlates of establishing, maintaining and switching brain states. Trends Cogn. Sci. 16, 330–337 10.1016/j.tics.2012.05.001 22613871 PMC3419378

[B84] Van Ede F. de Lange F. Jensen O. Maris E. (2011). Orienting attention to an upcoming tactile event involves a spatially and temporally specific modulation of sensorimotor alpha- and beta- band oscillations. J. Neurosci. 31, 2016–2024 10.1523/JNEUROSCI.5630-10.2011 21307240 PMC6633042

[B85] Van Essen D. C. Dickson J. Harwell J. Hanlon D. Anderson C. H. Drury H. A. (2001). An integrated software system for surface-based analyses of cerebral cortex. J. Am. Med. Inform. Assoc. 8, 443–459 10.1136/jamia.2001.0080443 11522765 PMC131042

[B86] Varela F. Lachaux J. P. Rodriguez E. Martinerie J. (2001). The brain web: phase synchronization and large scale integration. Nat. Rev. 2, 229–239 10.1038/35067550 11283746

[B87] Vincent J. L. Kahn I. Snyder A. Z. Raichle M. E. Buckner R. L. (2008). Evidence for a frontoparietal control system revealed by intrinsic functional connectivity. J. Neurophysiol. 100, 3328–3342 10.1152/jn.90355.2008 18799601 PMC2604839

[B88] Wallace B. A. Shapiro S. L. (2006). Mental balance and well-being: building bridges between Buddhism and Western psychology. Am. Psychol. 61, 690–701 10.1037/0003-066x.61.7.690 17032069

[B89] Watanabe T. Hirose S. Wada H. Imai Y. Machida T. Shirouzu I. (2013). A pairwise maximum entropy model accurately describes resting-state human brain networks. Nat. Commun. 4:1370 10.1038/ncomms2388 23340410 PMC3660654

[B90] Xu J. Vik A. Groote I. R. Lagopoulos J. Ellingsen O. Häberg A. K. (2014). Non directive meditation activates defaults mode network and areas associated with memory retrieval and emotional processing. Front. Hum. Neurosci. 8:86 10.3389/fnhum.2014.00086 24616684 PMC3935386

[B91] Zanesco A. P. King B. G. MacLean K. A. Saron C. D. (2013). Executive control and felt concentrative engagement following intensive meditation training. Front. Hum. Neurosci. 7:566 10.3389/fnhum.2013.00566 24065902 PMC3776271

